# Articles You Might Have Missed

**DOI:** 10.1007/s13181-025-01079-4

**Published:** 2025-05-22

**Authors:** Allison R. Font, Michael O. Khoury, Christopher D. Riviello, Michael F. Singer

**Affiliations:** Department of Emergency Medicine, Division of Medical Toxicology, Jefferson Einstein Hospital, Philadelphia, PA USA

**Keywords:** Snakebite infections, GLP-1 agonists for substance use disorders, Toluene, BK channels, NaV1.8, Non-opioid analgesic

**Article Title #1:** Bonilla-Aldana DK, Bonilla-Aldana JL, Ulloque-Badaracco JR, et al.: Snakebite-associated infections: a systematic review and meta-analysis. *American Journal of Tropical Medicine and Hygiene.* 2024;110(5):874–886.

**Background**: In many developing countries, snakebites still represent a large public health concern. While envenomation may cause substantial morbidity and mortality, the bite itself may pose secondary risks, including infection.

**Research Questions**: What is the prevalence of snakebite infections and what are the bacteria isolated from these infections?

**Methods**: A systematic review of published data was performed using the Preferred Reporting Items for Systematic Reviews and Meta-Analyses (PRISMA) guidelines. Studies were chosen from PubMed (NLM), Scopus (Elsevier), Web of Science (Clarivate), SciELO, and Embase (Elsevier). Studies included cohort, case–control or cross-sectional studies that assessed the prevalence of any of the following: infections in snakebites, the different bacteria identified, or surgical intervention secondary to snakebite infection. There were no exclusions based on age of subjects. All studies included were evaluated for bias using either the Newcastle–Ottawa Scale (NOS) or the adapted NOS.

**Results**: A total of 62 studies (from 525 identified articles) were included (n = 84,296 subjects). These studies were conducted between 1989 and 2022 and on the following continents: Asia (33 studies), the Americas (24 studies), Africa (four studies), and Europe (one study). All studies defined the diagnosis of snakebite based on physical examination and snakebite infection based on signs of an infected wound or progressive tissue necrosis at or around the bite site. Overall, the prevalence of snakebite infection was 27% (95% CI: 22.0–32.0%). In subgroup analysis by continent the prevalence of snakebite infection was 32% in Asia, 21% in the Americas, and 29% in Africa. In snake family subgroup analysis, the prevalence of infection by Elapids and Vipers was 62% and 31%, respectively. Prevalence of Gram-positive or Gram-negative (assessed in 16 studies) and anaerobic bacteria (assessed in eight studies) was 40.0%, 63.0% and 4.0%, respectively. Prevalence of isolated bacteria was: *Morganella morganii* (32.0%), *Enterococcus* sp (23.0%), *Staphylococcus aureus* (15.0%), *Proteus* sp (8.0%), *Shewanella* sp (7.0%), *Eschericia coli* (6.0%), *Citrobacter* spp (5.0%), *Bacteroides fragilis* (5.0%), *Serratia* spp (5.0%), *Aeromonas hydrophil* (5.0%), *Pseudomonas aeruginosa* (4.0%), and *Klebsiella pneumonia* (3.0%).

**Conclusion**: There is a significant prevalence of snake-bite associated infections, more commonly involving Elapid bites and caused by different organisms, primarily *M. morganii*.

**Critique**: The main limitation was standardized criteria for bite-related infection. This study did not assess which snake species were more prone to cause infections or the specific bacteria most commonly found in the mouths of individual snake species. The clinical relevance of all cultured bacteria (i.e., was it pathological) was unknown. The bite location (e.g., leg), severity of envenomation, patient comorbidities, time interval between bite and medical care, and choice of antibiotics were not reported.

**Implication for Toxicologists**: Secondary morbidity from snake bites, including local infection, should be looked for and discussed with patients.

**Article Title #2:** Shen MR, Owusu-Boaitey K, Holsen LE, et al.: The efficacy of GLP-1 agonists in treating substance use disorder in patients: a scoping review. *Journal of Addiction Medicine*. 2024;18(5):488–498.

**Background**: Glucagon-like peptide-1 (GLP-1) receptor agonists have been proposed as potential treatments for substance use disorders (SUD) due to their effects on the mesolimbic regions in the brain. The proposed mechanism involves blunted dopamine release in reward circuits and reduced responsiveness to addiction triggers.

**Research Question**: What is the efficacy, tolerability, and safety of using GLP-1 agonists for SUD treatment?

**Methods**: The authors developed a search algorithm to assess PubMed and APA PsychINFO from inception to October 19, 2023, using the preferred Reporting Items for Systematic Reviews and Meta-Analysis (PRISMA) guidelines. Included articles were peer-reviewed manuscripts written in English that discussed GLP-1 agonists in the treatment of SUDs in human subjects. Case reports, case series and study protocols were excluded. Two authors independently screened manuscripts and extracted study population, design, intervention (dosing, route, adjuncts), and outcomes, stratified by body mass index. A third senior author resolved disagreements.

**Results**: A total of 308 studies were identified but only five met inclusion criteria; two investigated tobacco use disorder (TUD), two studied alcohol use disorder (AUD), and one studied cocaine use disorder (CUD). The first TUD study evaluated 84 patients with obesity or prediabetes who received a 21-mg nicotine patch and smoking cessation counseling; the experimental group also received weekly subcutaneous injections of 2 mg exenatide. The experimental group exhibited significantly greater smoking abstinence at six weeks (46.3% vs 26.8%; RR, 1.70), lower tobacco cravings at the end of treatment, and lower post-cessation body weight. The second TUD study evaluated 244 patients by comparing weekly 1.5 mg dulaglutide SQ to placebo with both groups receiving varenicline 2 mg PO and behavioral counseling. They found no significant difference in tobacco abstinence at 12 weeks (63% placebo vs 65%; difference − 1.9%; 95% CI, − 10.7 to 14.4; P = 0.86). However, dulaglutide reduced post-cessation weight gain (− 2.9 kg; 95% CI, − 3.6 to − 2.3; P < 0.001) and hemoglobin A1c (− 0.25%; IQR, − 0.36 to − 0.14; P < 0.001) compared to placebo.

The first AUD study evaluated 127 participants who received weekly 2 mg SQ exenatide or placebo, along with behavioral treatment. Exenatide was not superior to placebo at 26 weeks or 6 months post-trial. In participants with obesity (BMI > 30), exenatide reduced heavy drinking days by 23.6% (95% CI, − 44.4 to − 2.7; P = 0.034) and total alcohol intake. However, among those with BMI < 25, exenatide increased heavy drinking days by 27.5% (95% CI, 4.7 to 50.2; P = 0.024) without significantly changing total alcohol intake. The second AUD study compared 38,484 new GLP-1 agonist users to 49,222 users of DPP-4 inhibitors from the Danish National Prescription Registry. GLP-1 agonist initiation was associated with a lower risk of an alcohol-related event (hazard ratio = 0.46; 95% CI, 0.24–0.86) at three months and one year.

Angarita et al. conducted a trial in 13 hospitalized adults with CUD, administering a single 5 mcg subcutaneous dose of exenatide or placebo prior to two cocaine self-administration sessions. Exenatide did not significantly affect self-administered cocaine (8.5 administrations ± 1.2 vs. 9.1 ± 1.2; p = 0.39), euphoria (VAS 4.4 ± 0.8 vs. 4.1 ± 0.8; p = 0.21), or craving (VAS 5.6 ± 0.9 vs. 5.4 ± 0.9; p = 0.46).

Overall, there were limited reports of adverse medication events to the GLP-1 agonists with most noted in the TUD study where 10 subjects withdrew due to gastrointestinal symptoms.

**Conclusion**: There are limited and mixed results for the use of GLP-1 agonists on SUDs. More work involving larger subject groups is required.

**Critique**: These were very few studies that used different GLP-1 agonists with varying homology to endogenous GLP-1 that may influence effectiveness in SUDs. Several studies combined GLP-1 agonists with standard treatments, which may have obfuscated small effects. Follow-up periods were often short.

**Implication for Toxicologists**: Toxicologists should be familiar with the limited evidence for using these agents for SUDs, as well as the potential complications.

**Article Title #3:** Shaw AA, Steketee JD, Bukiya AN, et al.: Toluene is a cerebral artery constrictor acting via BK channels. *Neuropharmacology.* 2025;266: 110272.

**Background**: The chronic effects of toluene misuse are well-described but less is known about the molecular targets mediating acute intoxication. Prior studies have demonstrated overlapping features of toluene intoxication and central nervous system (CNS) hypoperfusion, particularly in territories perfused by the middle cerebral artery (MCA). A critical mediator of cerebral perfusion is the large-conductance Ca^2+^ activated potassium (BK) channels in vascular smooth muscle. This may serve as a potential mechanism for acute toluene intoxication.

**Research Questions**: Does toluene exposure that resembles recreational misuse reduce MCA diameter and cause CNS hypoperfusion? Is this inhibition via BK channels independent of endothelial factors?

**Methods**: Cerebral artery diameter was measured in vivo in wild-type rats following cranial window surgery, allowing visualization of pial arteries (MCA branches) during exposure to toluene vapor. To assess toluene’s effect on endothelium-derived factors, MCAs segments from wild-type rats and mice were de-endothelialized and exposed to toluene. Wild-type and BK channel knockout (KCNMA1^−/−^) mice were also studied. To rule out non-BK channel mediated effects, de-endothelialized MCAs were exposed to 1 mM toluene, 1 µM paxilline (a BK channel antagonist), or both. Inside-out patch clamp recordings were performed on smooth muscle cells from wild-type rat MCAs; channel activity and conductance were compared.

**Results**: In vivo toluene demonstrated dose-dependent MCA vasoconstriction that became evident at concentrations ≥ 3,000 ppm and reached maximal effect (EC_100_) at concentrations seen with recreational use (around 8,000 ppm); EC_100_ males: 8,634 ± 454, females: 7,754 ± 248 ppm. Mean MCA diameter decreased by approximately 8% in both sexes, corresponding to a 36% reduction in cerebral blood flow. This would impair oxygen delivery to MCA territories. Vasoconstrictive effects became evident at a toluene blood level of > 100 ppm and maximal at concentrations exceeding 1,000 ppm.

In an ex vivo model, toluene constricted MCA segments in de-endothelialized segments with similar concentration dependence when compared to endothelium-intact specimens (i.e., reduced by 5.5% and 3.3% for male and female rats, respectively). This suggests toluene’s ability to cause cerebral vasoconstriction was independent of endothelium-derived factors.

Patch clamp testing using toluene decreased BK channel steady state activity to 88 ± 4% compared to control (p = 0.0086). Additionally, application of toluene significantly reduced unitary current amplitude to 80.9 ± 1.2% of control (p = 0.02381). These findings may indicate a disruption with gating and/or ion conduction that collectively suggest reduction in BK channel current. In the ex vivo de-endothelialized MCA specimens, toluene was unable to elicit additional vasoconstriction in the presence of paxilline, a known BK channel antagonist. This suggests toluene’s effect is mediated through this same channel.

**Conclusion**: Toluene concentrations similar to those experienced during intentional misuse inhibit BK channels, independent of endothelium-derived factors, causing vasoconstriction and suspected CNS hypoperfusion.

**Critique**: Limitations included prolonged toluene exposures which may not mimic real-world abuse patterns. Additionally, toluene effects on surrounding neural elements were not evaluated and may contribute to cerebral vasoconstriction.

**Implication for Toxicologists**: This study offers objective findings that can be used to educate about the dangers of toluene misuse. These findings may provide future insights for further investigation into other solvents and targeted management.

**Article Title #4:** Osteen JD, Immani S, Tapley TL, et al.: Pharmacology and mechanism of action of suzetrigine, a potent and selective Na_V_1.8 pain signal inhibitor for the treatment of moderate to severe pain. *Pain Therapy.* 2025;14:655–674.

**Background**: Subtypes of the voltage-gated sodium channels, particularly Na_V_1.8, are potential targets for analgesic treatments because they are primarily expressed in peripheral pain-sensing neurons rather than the central nervous system (CNS). Targeting these receptors may offer pain relief without typical CNS-related side effects and addiction risks.

**Research Questions**: Does selective inhibition of the Na_V_1.8 channel with suzetrigine (VX-548) provide effective analgesia in moderate to severe pain? Is this treatment safe and without addiction risk?

**Methods**: The study employed both preclinical and clinical methodologies to evaluate the efficacy, safety, and abuse potential of suzetrigine. Preclinical assessments included electrophysiological testing via patch-clamp techniques. Radiolabeled binding assays evaluated the drug's specificity for the voltage-sensing domain 2 (VSD2) of the Na_V_1.8 channel. Computational Tanimoto similarity analysis was used to assess chemical resemblance to known substances of abuse. Additional pharmacology assays screened for off-target interactions across various receptors and ion channels, while gene expression analysis confirmed the lack of SCN10A (Na_V_1.8) expression in the CNS. Animal studies in rats and monkeys evaluated the compound's effects on the CNS, cardiovascular, and respiratory systems, as well as its potential for addictive behavior. Additionally, Phase 3 clinical trials were discussed, which enrolled over 2,400 patients experiencing acute pain. These trials aimed to assess the clinical effectiveness of suzetrigine, monitor adverse effects, and evaluate the risk of abuse or addiction in human populations.

**Results**: Suzetrigine shows selectivity for the Na_V_1.8 sodium channel, with over 31,000-fold specificity compared to other subtypes. Radiolabeled binding assays confirmed high-affinity binding to VSD2 of Na_V_1.8. Protein expression validated channel integrity and Tanimoto analysis showed low similarity to drugs of abuse. Broad pharmacology screening revealed minimal off-target effects, and gene expression data confirmed little to no CNS Na_V_1.8 expression. Animal studies found no major CNS, cardiovascular, or respiratory side effects, and no signs of addiction. In vitro models confirmed that suzetrigine stabilizes Na_V_1.8 in a closed state, dampening pain signals. Phase 3 trials showed effective pain relief with minimal CNS impact and no abuse potential.

**Conclusion**: Suzetrigine is a selective Na_V_1.8 inhibitor with potential use in pain management and a favorable safety profile. The evidence suggests minimal or no risk for abuse or addictive potential.

**Critique**: The clinical trial durations were relatively short (up to 14 days) providing no data about long-term efficacy and safety. More work concerning the long-term effects, particularly in humans, is required. The authors all work for the pharmaceutical company marketing suzetrigine.

**Implication for Toxicologists**: Suzetrigine may represent an important advancement in pain management. More research is required.

